# Closed‐incision negative pressure therapy at −125 mmHg significantly reduces surgical site complications following total hip and knee arthroplasties: A stratified meta‐analysis of randomized controlled trials

**DOI:** 10.1002/hsr2.425

**Published:** 2022-01-23

**Authors:** Kareem G. Elhage, Mohamed E. Awad, Furqan B. Irfan, Joshua Lumbley, Gamal Mostafa, Khaled J. Saleh

**Affiliations:** ^1^ FAJR Scientific Northville Michigan 48167 USA; ^2^ Wayne State University, School of Medicine Detroit Michigan USA; ^3^ NorthStar Anesthesia‐Detroit Medical center Detroit Michigan USA; ^4^ Michigan State University, College of Osteopathic Medicine Detroit Michigan USA; ^5^ Surgical Outcomes Research Institute, John D. Dingell VA Medical Center Detroit Michigan USA

**Keywords:** conventional dressing, negative pressure wound therapy, surgical site complications, total hip arthroplasty, total knee arthroplasty

## Abstract

**Background:**

Closed‐incision negative pressure wound therapy (ciNPT) has shown promising effects for managing infected wounds. This meta‐analysis explores the current state of knowledge on ciNPT in orthopedics and addresses whether ciNPT at −125 mmHg or −80 mmHg or conventional dressing reduces the incidence of surgical site complications in hip and knee arthroplasty.

**Methods:**

This meta‐analysis was conducted according to the Preferred Reporting Items for Systematic Review and Meta‐analysis (PRISMA) guidelines and Cochrane Handbook. Prospective randomized controlled trials (RCTs) with ciNPT use compared to conventional dressings following hip and knee surgeries were considered for inclusion. Non‐stratified and stratified meta‐analyses of six RCTs were conducted to test for confounding and biases. A *P* value less than .05 was considered statistically significant.

**Results:**

The included six RCTs have 611 patients. Total hip and knee arthroplasties were performed for 51.7% and 48.2% of the included population, respectively. Of 611 patients, conventional dressings were applied in 315 patients and 296 patients received ciNPT. Two ciNPT systems have been used across the six RCTs; PREVENA Incision Management System (−125 mmHg) (63.1%) and PICO dressing (−80 mmHg) (36.8%). The non‐stratified analysis showed that the ciNPT system had a statistically significant, lower risk of persistent wound drainage as compared to conventional dressing following total hip and knee arthroplasties (OR = 0.28; *P* = .002). There was no difference between ciNPT and conventional dressings in terms of wound hematoma, blistering, seroma, and dehiscence. The stratified meta‐analysis indicated that patients undergoing treatment with high‐pressure ciNPT (120 mmHg) displayed significantly fewer overall complications and persistent wound drainage (*P* = .00001 and *P* = .002, respectively) when compared to low‐pressure ciNPT (80 mmHg) and conventional dressings. In addition, ciNPT is associated with shorter hospital stays. (*P* = .005).

**Conclusion:**

When compared to conventional wound dressing and −80 mmHg ciNPT, the use of −125 mmHg ciNPT is recommended in patients undergoing total joint arthroplasty.

## Graphical abstract

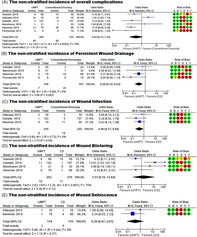

## 1 INTRODUCTION

Surgical site complications (SSCs)— including delayed incision healing, prolonged incision drainage, seromas and hematomas, incision abscesses, and surgical site infections (SSIs)—are potential adverse outcomes occurring after orthopedic surgeries. In chronic cases, this can cause physical, mental, or emotional disability.^1^ Between the years of 1993 and 2005, the need for THA and TKA in the United States almost doubled.^2^ During a similar 10‐year period in Canada, there was an 86.6% increase.^3^ A significant increase in the need for THA and TKA between the years of 2005 to 2012 was also observed in Europe.^4^ As a result, patient hospital stays increased, which in turn raised healthcare costs.^5^ In 2017, the Centers for Diseases Control and Prevention (CDC) published guidelines for the prevention of surgical infection on the basis of a literature search and review of studies published from 1998 to 2014.^6^ Recommendations included that patients should shower or bathe with soap or an antiseptic wash before surgery. Antimicrobial prophylaxis is advised and might be administered in a timed modality for different surgical incisions, such as adding a bactericidal agent at the time of the incision. The implementation of perioperative glycemic control is advised; using blood glucose target levels less than 200 mg/dL is advised as diabetes is a significant risk factor for SSI.^7^

Guidelines are uncertain for prosthetic joint arthroplasty, with the exception of blocking the use of additional antimicrobial treatment in the operating room (OR) after incisions are closed. Past protocols have used several perioperative and postoperative procedures to avoid SSCs.^8^ Open wounds have been treated with a cotton gauze dressing soaked with a variety of antiseptic solutions, such as Lavasept 0.04%, hydrogen peroxide 3%, Octenisept 0.1%, povidone‐iodine 10%, and chlorhexidine digluconate 20%, to decrease bacterial load during total joint arthroplasty (TJA).^6^, ^9^ Various types of wound dressings have been used, including glue‐based and hydrofiber dressings with or without silver impregnation. In particular, a silver hydrofiber dressing (Aquacel Ag Surgical Dressing, ConvaTec, Princeton, NJ) has been studied for its effectiveness to prevent infection.^10^ Antibiotic‐loaded bone cement (ALBC) has wide use in Europe^8^ but remains controversial in the United States of America due to potential resistance and toxicity of antibiotics.^11^ The Food and Drug Administration (FDA) approved only six low‐dose ALBCs to be used in the second stage surgery of a two‐stage exchange for periprosthetic joint infection (PJI).^11^

In orthopedics, closed‐incision negative pressure therapy (ciNPT) was first documented in 2006.^12^ It was recommended for use in patients at risk of developing postoperative incision infection in 2016 by an international multidisciplinary consensus.^13^ Saleh et al found that postoperative drainage at 5 days or greater was associated with a 12.7 times increased likelihood to develop PJI in patients undergoing knee and hip arthroplasties.^14^ Several studies have shown the benefits of ciNPT in patients undergoing primary elective hip and knee arthroplasties.^15^, ^16^, ^17^ Reduction in SSI, hospital stays, hematoma and seroma formation, and healthcare costs have been observed in trauma arthroplasty and spinal fractures.^16^, ^17^, ^18^ Few different portable ciNPT systems that have been commercially available. Both systems are disposable battery powered device with an absorbent layer‐based peel‐and‐place dressing primarily designed for closed incisions. However, these systems vary in the applied pressure (at −125 mmHg vs −80 mmHg) and design characteristics. Although there is current solid evidence that supports the superiority of any of these systems, there are few randomized controlled trials comparing each ciNPT system with conventional dressings. Further studies are needed to assess the efficacy of ciNPT and stratify the outcome based on the applied pressure. Thus, the aim of this study was to perform a meta‐analysis exploring the current state of knowledge on the application of ciNPT in orthopedics and to address whether ciNPT at −125 mmHg or −80 mmHg or conventional dressing reduces the incidence of surgical site complications in hip and knee arthroplasty.

## 2 MATERIALS AND METHODS

### 2.1 Protocol

This systematic review and meta‐analysis were conducted according to the preferred Reporting Items for Systematic Review and Meta‐analysis (PRISMA) guidelines and the Cochrane Handbook.^19^ This meta‐analysis is exempt from ethics approval because we collected and synthesized data from previous clinical trials in which informed consent has already been obtained by the trial investigators.

### 2.2 Literature search

A detailed comprehensive literature search was performed twice in PubMed, Web of Science, and EMBASE in the time period between January and August 2020. The following keywords were used: (“negative pressure wound therapy” OR “negative pressure therapy” OR “vacuum‐assisted closure” OR “NPWT”) OR “closed incision negative pressure wound therapy” OR “closed incision negative pressure therapy”) AND (“Arthroplasty, Replacement, Knee” OR “Arthroplasty, Replacement, Hip”[Mesh] AND “total knee”[All Fields] OR “total hip”[All Fields]. Time frame keywords were not included in search terms.

### 2.3 Eligibility criteria

Exclusion and inclusion were derived from PICO [Population, Intervention, Comparison and Outcome] and non‐PICO‐based exclusion taxonomy (eg, language, article not available or duplicate data/study). All prospective randomized controlled trials (RCTs) published in the English language, regardless of number of patients investigating the use of ciNPT following hip and knee surgeries, as compared to conventional dressings were considered for inclusion. Exclusion criteria applied were studies in languages other than English, meta‐analysis studies, pre‐clinical studies (ie, animal or bench studies), veterinary studies, conference abstracts, reviews, expert opinions, protocols; non‐clinical reports, and unpublished studies.

PICO criteria:

- Population: All RCTs, prospective non‐randomized, and retrospective cohort studies that included patients underwent Hip and knee surgeries.
- Intervention: ciNPT
- Comparison; conventional dressings
- Outcome:
  - Primary outcomes:
    - Non‐stratified and stratified meta‐analysis of the incidence of the following:
      - Overall complications
      - Persistent wound drainage
      - Wound infection
      - Wound blistering
      - Wound dehiscence
      - wound seroma
      - Wound hematoma
  - Secondary outcomes:
    - Re‐admission rate
    - Length of hospital stay (LOHS)

### 2.4 Study selection

Two independent reviewers screened abstracts and manuscripts derived by the search and selected eligible papers based on the eligibility criteria. Some articles were excluded by reviewing the inclusion criteria in the title or abstract. All other studies required full text review in order to determine relevance.

### 2.5 Data glean from eligible studies

Two reviewers (KE and MEA) extracted information from all eligible publications independently. A data collection sheet was established to sort quantitative and qualitative information for analysis. The data were extracted using the following variables: (a) demographics and characteristics (author, country of trial, year of publication, patients number, age, sex, and BMI). (b) surgery characteristics (type of surgery, type of anesthesia, and postoperative drain); and (c) intervention characteristics (type of system, pressure, conventional dressing, and therapy duration). In addition, primary outcome variables (incidence of infection, blistering, dehiscence, seroma, bruising, hematoma, persistent wound drainage, drop in hemoglobin level, and transfusion rate) and secondary outcomes (length of hospital stay and rate of readmission) were also extracted (Table 1).

**TABLE 1:** Demographic data, surgery type, intervention characteristics, and variables of included studies

Author/reference	Country	Groups	Dressings	Pressure (mmHg)	Mean duration	N	Age (mean)	BMI (mean)	Sex		Surgery	ASA classification			N of comorbidities (mean)	Anesthetic			Drain used	Wound closure			Anti‐coagulation protocol
									F	M		1	2	3		GA	SA	other		Sutures	Staples	others	
Gillespie^20^	Australia	CD	Hydrocolloid dressing			35	62.5	29.8	18	17	primary THA‐DAA	2	22	11	3	30	8	1	5	13	23	17	NA
		ciNPT	PICO dressing (Smith & Nephew, Hull, UK),	80 mmHg	5	35	63.8	29.9	15	20		2	17	16	3	29	7	1	5	13	23	2	
Karlakki^16^	UK	CD	Conventional dressing (Mepore or Tegaderm)			107	69.2	28.4	52	55	Primary TJA	24	69	11	1.7	58	46	3	47	34	73	0	Enoxaparin; THA (4 weeks)/TKA (2wk)
		ciNPT	PICO dressing (Smith & Nephew, Hull, UK)	80 mmHg	7	102	69	30.1	53	49		23	60	11	1.9	53	47	2	50	26	76	0	
Newman^21^	USA	CD	AQUACEL Ag			80	65	33.4	35	45	TJA	10		70		70	10	0	NA	NA			NA
		ciNPT	PREVENA system (KCI, San Antonio, USA).	125 mmHg	>2	79	65	31.9	39	40		13		66		70	9	0	NA	NA			
Giannini^22^	Italy	CD	Povidone‐iodine gauze and patch wound dressing			50	66.8	28.2	32	18	Revision TJA	NA			1.7	NA			50	NA			NA
		ciNPT	PICO dressing (Smith & Nephew, Hull, UK),	80 mmHg	7	50	66	27.7	31	19		NA			2.3	NA			50	NA			
Manoharan^23^	Australia	CD	Dry dressing			33	66	29.79	14	19	Primary TKA	NA			NA	NA			33	NA			Chemical and Mechanical prophylaxis
		ciNPT	PREVENA system (KCI, San Antonio, USA).	125 mmHg	8	21						NA			NA	NA			21	NA			
Pachowsky^24^	Germany	CD	Dry dressing			10	70.5	NA	NA	NA	Primary THA	NA			NA	NA			NA	NA			NA
		ciNPT	VAC Therapy, (KCI, San Antonio, USA).	125 mmHg		9	66.22	NA	NA	NA		NA			NA	NA			NA	NA			

Abbreviations: ASA, American society of anesthesiologists; BMI, body mass index; CD, conventional dressing; ciNPT, closed‐incision negative pressure therapy; THA‐DAA, total hip arthroplasty‐direct anterior approach; TJA, total joint arthroplasty.

### 2.6 Risk of bias assessment of randomized controlled trials

We used the Cochrane collaboration's assessment tool for risk of bias^19^ to assess the methodological bias of included RCTs. The following items that were assessed included the randomization, allocation concealment, blinding, incomplete outcome data (attrition bias), and selective reporting (reporting bias).

### 2.7 Synthesis of results

We used Prism (version 5.0.0, graphpad) to analyze the data. We reported descriptive statistics including the mean, SD, range, and median. We used a Pearson correlation coefficient for normally distributed data. Mean differences with their corresponding 95% confidence intervals (CIs) were generated for continuous outcome data, and risk ratios (RRs) with 95% CIs were generated for dichotomous data. A *P* value less than .05 was considered statistically significant. *I* ^2^ values were calculated to estimate the heterogeneity among the included studies. In the presence of homogeneity (*I* ^2^ < 50%), the fixed effects model was used to estimate the overall effects. If there was significant heterogeneity among included studies, the random effects model was used. The meta‐analysis was undertaken using RevMan 5.3 software. We analyzed sensitivity and specificity by using an online tool (MedCalc Diagnostics, MedCalc software).

## 3 RESULTS

### 3.1 Literature search and study selection

**FIGURE 1:**
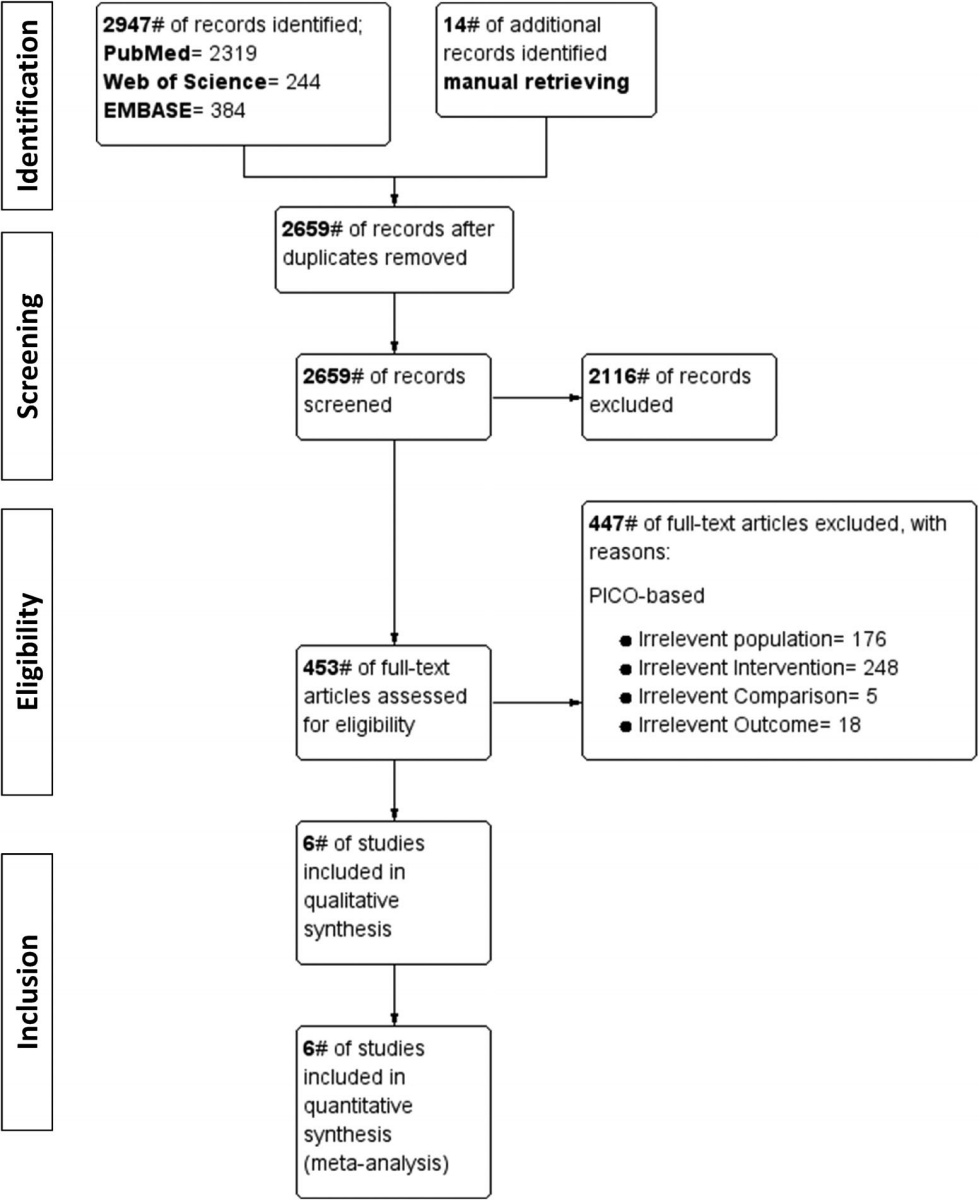
Flowchart showing search strategy and study identification, inclusion, and exclusion

Our search strategy identified 2947 publications for possible meta‐analysis inclusion. A manual search through the screened publications produced an additional 14 articles. After duplicates were removed, 453 full‐text articles were screened for eligibility to the inclusion criteria. After screening, 447 studies were excluded due to irrelevant population (n = 176), intervention (n = 248), comparison (n = 5), or outcome (n = 18). After eligibility screening, six RCTs met the inclusion criteria (Figure 1).

### 3.2 Characteristics of included studies

**FIGURE 2:**
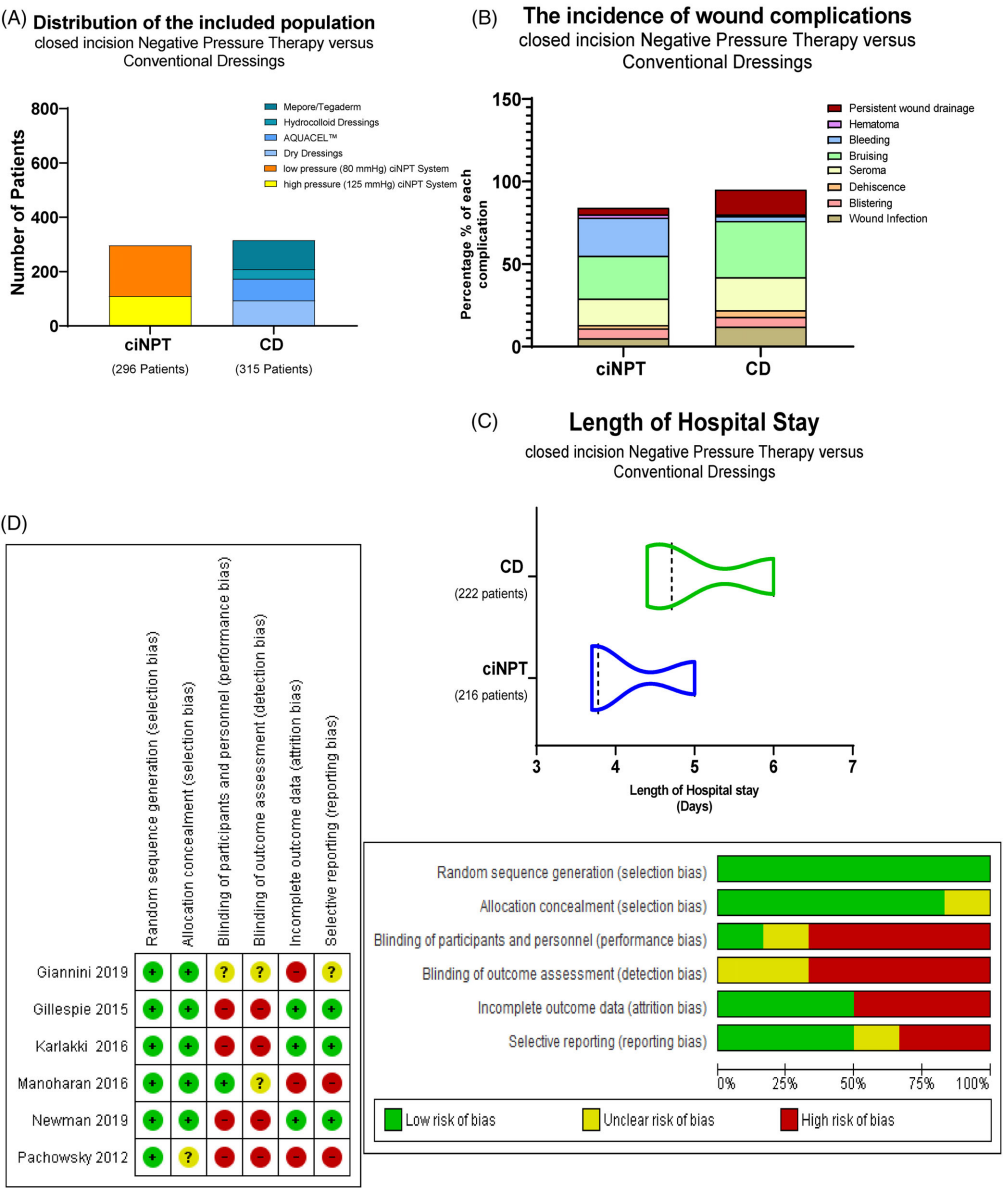
(A) Bar graphs showing characteristics of included studies including distribution of the included population, (B) incidence of wound complications, (C) length of hospital stay, (D) risk of bias assessment of randomized control trials (RCTs)

The six included RCTs assessed 611 patients. Total hip and knee arthroplasties were performed for 51.7% and 48.2% of the included population, respectively. The mean age across the included studies was 66 years old, ranging from 62.5 to 70.5 years. Fifty‐two percent of the included population were female across the RCTs. The average body mass index (BMI) was 29.8 across the studies. Of the 611 patients, conventional dressings were applied in 315 patients, and 296 patients received ciNPT. Two ciNPT systems were used across the six RCTs; PREVENA Incision Management System (KCI, San Antonio, TX) (−125 mmHg) (63.1%) and PICO Single Use Negative Pressure Wound Therapy (Smith & Nephew, Hull, UK) (−80 mmHg) (36.8%). In three RCTs, the conventional dressing used was dry sterile dressing (DSD). Hydrocolloid dressing, Mepore/Tegaderm, and Aquacel (ConvaTec, Princeton, NJ) were represented in the other three RCTs^16^, ^20^, ^21^ (Figure 2A). The average length of hospital stays in ciNPT and conventional dressing (CD) groups was 4.16 ± 0.72 and 5.03 ± 0.84 days, respectively (Figure 2C). The incidence of complications within ciNPT and CD groups was 16.7% and 29.7%, respectively. The −125 mmHg ciNPT use was associated with lower rate of complications (15.5%), as compared to the −80 ciNPT use (23.8%) (Figure 2B).

### 3.3 Geographical distribution of the included studies

Six RCTs (611 patients) with study periods between 2012 and 2019 have compared ciNPT to CD following total hip and knee arthroplasties. A total of 33.3% (2/6)^20^, ^23^ of the studies were conducted in Australia, whereas the other four trials came from the United States (1/6),^21^ United Kingdom (1/6),^16^ Italy (1/6),^22^ and Germany (1/6).^24^

### 3.4 Risk of bias assessment of randomized controlled trials

Of the six RCTs, all studies reported the methodology of the random sequence generation either briefly or in detail.^16^, ^20^, ^21^, ^22^, ^23^, ^24^ However, allocation concealment was not clearly described. High‐performance and detection biases were noted across the six trials. Notably, blinding of participants and personnel was quite difficult. Regarding attrition bias, there were missing data and reported loss to follow‐up in half of the included studies^16^, ^20^, ^21^ (Figure 2D).

### 3.5 Sensitivity analysis

Using MedCalc, we calculated the sensitivity, specificity, and positive and negative likelihood ratios of both −125 mmHg and −80 mmHg compared to conventional dressings. The sensitivity of −125 mmHg ciNPT system as a tool to reduce the incidence of surgical site complications across the studies was 74.24%, and the specificity was 55.42%. However, the sensitivity of −80 mmHg ciNPT system across the studies was 54.93%, and the specificity was 50.32%, as compared to the conventional dressings (Tables 2 and 3).

**TABLE 2:** Sensitivity analysis of ciNPT at −125 mmHg

Sensitivity analysis of ciNPT at −125 mmHg		
Statistic	Value	95% CI
Sensitivity	25.76%	15.78% to 38.01%
Specificity	44.58%	36.87% to 52.48%
Positive likelihood ratio	0.46	0.30 to 0.72
Negative likelihood ratio	1.67	1.33 to 2.08

**TABLE 3:** Sensitivity analysis of ciNPT at −125 mmHg

Sensitivity analysis of ciNPT at −80 mmHg		
Statistic	Value	95% CI
Sensitivity	45.07%	33.23% to 57.34%
Specificity	49.68%	43.96% to 55.40%
Positive likelihood ratio	0.9	0.68 to 1.18
Negative likelihood ratio	1.11	0.87 to 1.40

## 4 PRIMARY OUTCOMES

### 4.1 The non‐stratified meta‐analysis of complications between ciNPT and conventional dressings

#### 4.1.1 Meta‐analysis of non‐stratified incidence of complications

**FIGURE 3:**
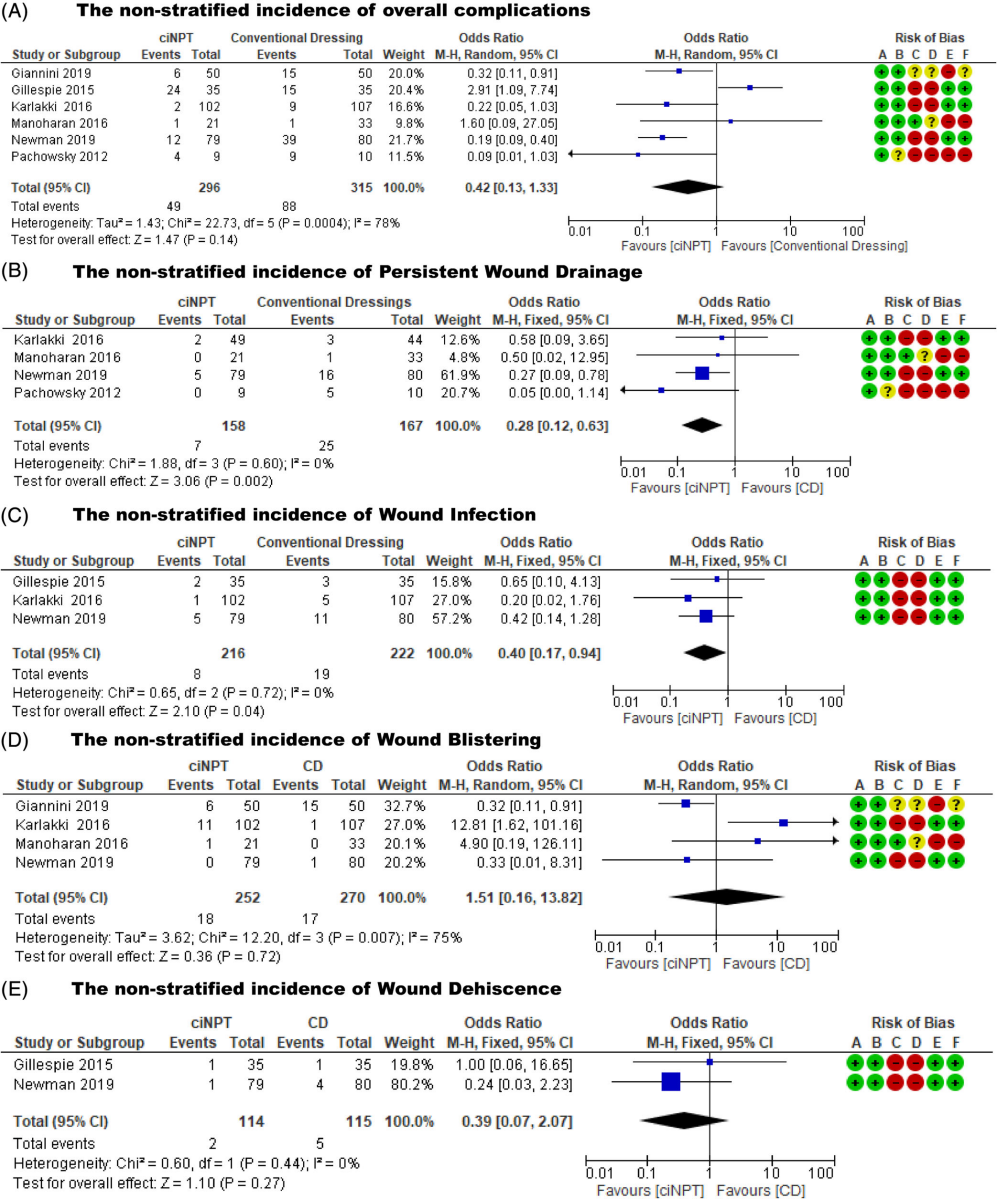
(A) Forest plots showing non‐stratified incidence of overall complications, (B) non‐stratified incidence of persistent wound drainage, (C) non‐stratified incidence of wound infection, (D) non‐stratified incidence of wound blistering, (E) non‐stratified incidence of wound dehiscence

All six studies^16^, ^20^, ^21^, ^22^, ^23^, ^24^ reported the incidence of complications. There was statistically significant heterogeneity in the studies (*P* = .0004; *I* ^2^ = 78%). Using the random effects model, the outcome results revealed that conventional dressings had a higher risk of complications as compared to ciNPT following total hip and knee arthroplasties. However, there was no statistically significant difference between groups (OR = 0.42; 95% CI: 0.13 to 1.33; *P* = .14) (Figure 3A).

#### 4.1.2 Meta‐analysis of the non‐stratified incidence of persistent wound drainage

Four studies,^16^, ^21^, ^23^, ^24^ which included 325 patients, reported the incidence of persistent wound drainage. There was no statistically significant heterogeneity in the studies (*P* = .60; *I* ^2^ = 0%). Using the fixed effects model, the outcome results revealed that the ciNPT system had a statistically significant lower risk of persistent wound drainage as compared to conventional dressing following total hip and knee arthroplasties (OR = 0.28; 95% CI: 0.12 to 0.63; *P* = .002) (Table 4 and Figure 3B).

**TABLE 4:** Significant results from non‐stratified (A) and stratified (B) meta‐analyses

Outcome	Number of Studies	Statistic Model	Difference between approaches			Heterogeneity	
			OR/MD	95% CI	*P* value	*I* ^2^ (%)	*P* value
Non‐stratified meta‐analysis of ciNPT vs conventional dressings							
Favors ciNPT system							
Persistent wound drainage	4	Fixed	0.28	0.12 to 0.63	.002**	0%	.60
Length of Hospital Stay	3	Fixed	−0.81	−1.37 to −0.24	0.005**	0%	.90
Stratified meta‐analysis of ciNPT based on the applied pressure							
Favors high pressure (‐125 mmHg ciNPT system)							
Overall complications	3	Fixed	0.20	0.10 to 0.41	<.00001***	21%	.28
Persistent wound drainage	3	Fixed	0.23	0.09 to 0.59	.002**	0%	.55

*Note*: Applies to all legends with forest plots.

Abbreviations: CD, conventional dressing; Chi^2^, chi‐square test; CI, confidence interval; ciNPT, closed‐incision negative pressure therapy; *I* ^2^, heterogeneity; M‐H, Mantel‐Haenszel test; OR, odds ratio, Tau^2^, variance.

** *P* ≤ .01,

*** *P* ≤ .001.

#### 4.1.3 Meta‐analysis of non‐stratified incidence of wound infection

Three studies,^16^, ^20^, ^21^ which included 438 patients, reported incidence of wound infection. There was no statistically significant heterogeneity in the studies (*P* = .72; *I* ^2^ = 0%). Using the fixed effects model, the conventional dressings had higher risk of wound infection as compared to ciNPT following total hip and knee arthroplasties. However, there was no statistically significant difference between groups (OR = 0.40; 95% CI: 0.17 to 0.94; *P* = .04) (Table 4 and Figure 3C).

#### 4.1.4 Meta‐analysis of non‐stratified incidence of wound blistering

Four studies,^16^, ^21^, ^22^, ^23^ which included 522 patients, reported wound blistering. There was statistically significant heterogeneity in the studies (*P* = .007; *I* ^2^ = 75%). Using the random effects model, the results revealed no statistically significant difference in the incidence of blistering between ciNPT and conventional dressing (OR = 1.51; 95% CI: 0.16 to 13.82; *P* = .72) (Table 4 and Figure 3D).

#### 4.1.5 Meta‐analysis of non‐stratified incidence of wound dehiscence

Two studies,^20^, ^21^ which included 229 patients, reported the incidence of wound dehiscence. There was no statistically significant heterogeneity in the studies (*P* = .44; *I* ^2^ = 0%). Using the fixed effects model, the outcome results revealed no statistically significant difference in the incidence of wound dehiscence between ciNPT and conventional dressing following total hip and knee arthroplasties (OR = 0.39; 95% CI: 0.07 to 2.07; *P* = .27) (Table 4 and Figure 3E).

#### 4.1.6 Meta‐analysis of non‐stratified incidence of wound seroma

**FIGURE 4:**
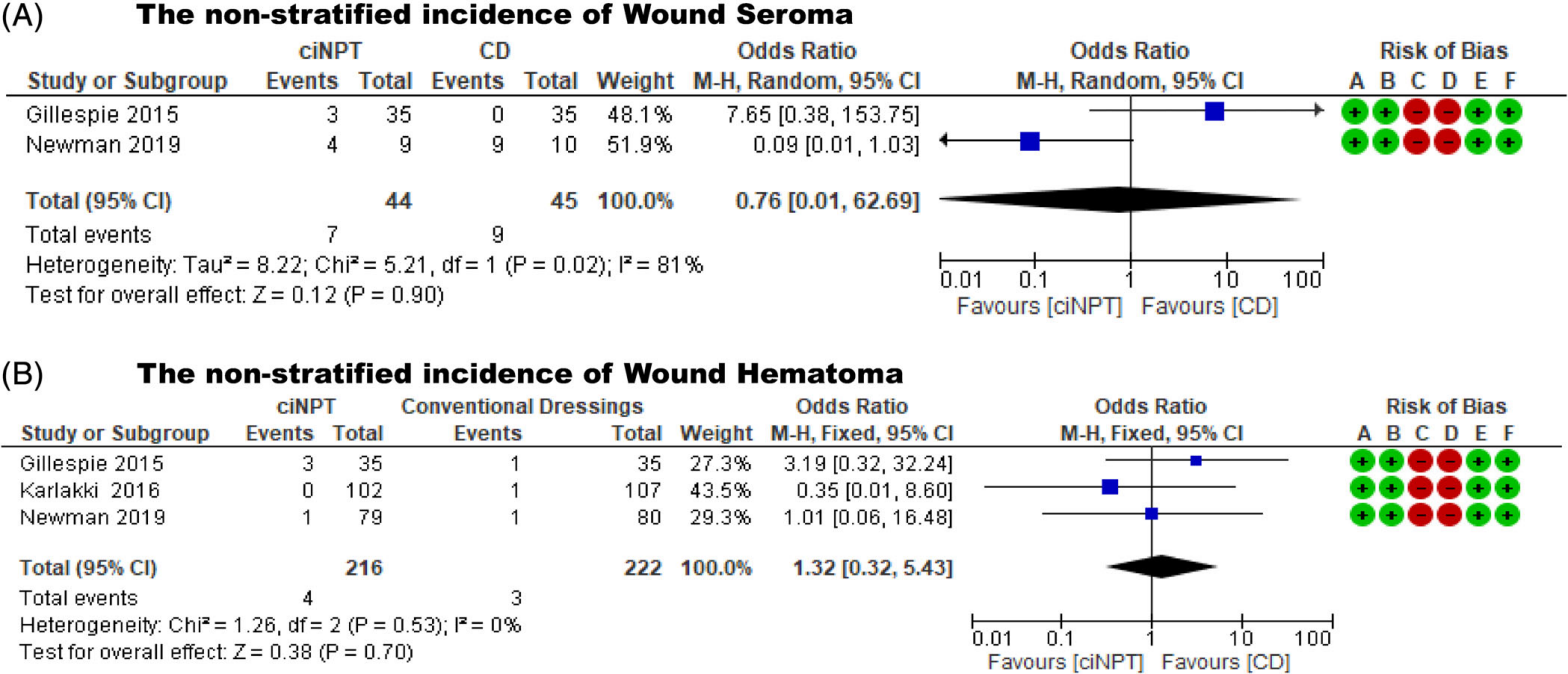
(A) Forest plots showing non‐stratified incidence of wound seroma, (B) non‐stratified incidence of wound hematoma

Two studies,^20^, ^21^ which included 89 patients, reported the incidence of wound seroma. There was no statistically significant heterogeneity in the studies (*P* = .02; *I* ^2^ = 81%). Using the random effects model, there was no statistically significant difference in the incidence of wound seroma between ciNPT and conventional dressing following total hip and knee arthroplasties (OR = 0.76; 95% CI: 0.01 to 62.69; *P* = .90) (Table 4 and Figure 4A).

#### 4.1.7 Meta‐analysis of non‐stratified incidence of wound hematoma

Three studies,^16^, ^20^, ^21^ which included 438 patients, reported the incidence of wound hematoma. There was no statistically significant heterogeneity in the studies (*P* = .53; *I* ^2^ = 0%). Using the fixed effects model, the results revealed no statistically significant difference in the incidence of wound hematoma between ciNPT and conventional dressing following total hip and knee arthroplasties (OR = 1.32; 95% CI: 0.32 to 5.43; *P* = .70) (Table 4 and Figure 4B).

### 4.2 The stratified meta‐analysis of complications between ciNPT and conventional dressings based on the applied pressure and type of surgery

#### 4.2.1 Meta‐analysis of stratified incidence of complications using the −125 mmHg ciNPT system

**FIGURE 5:**
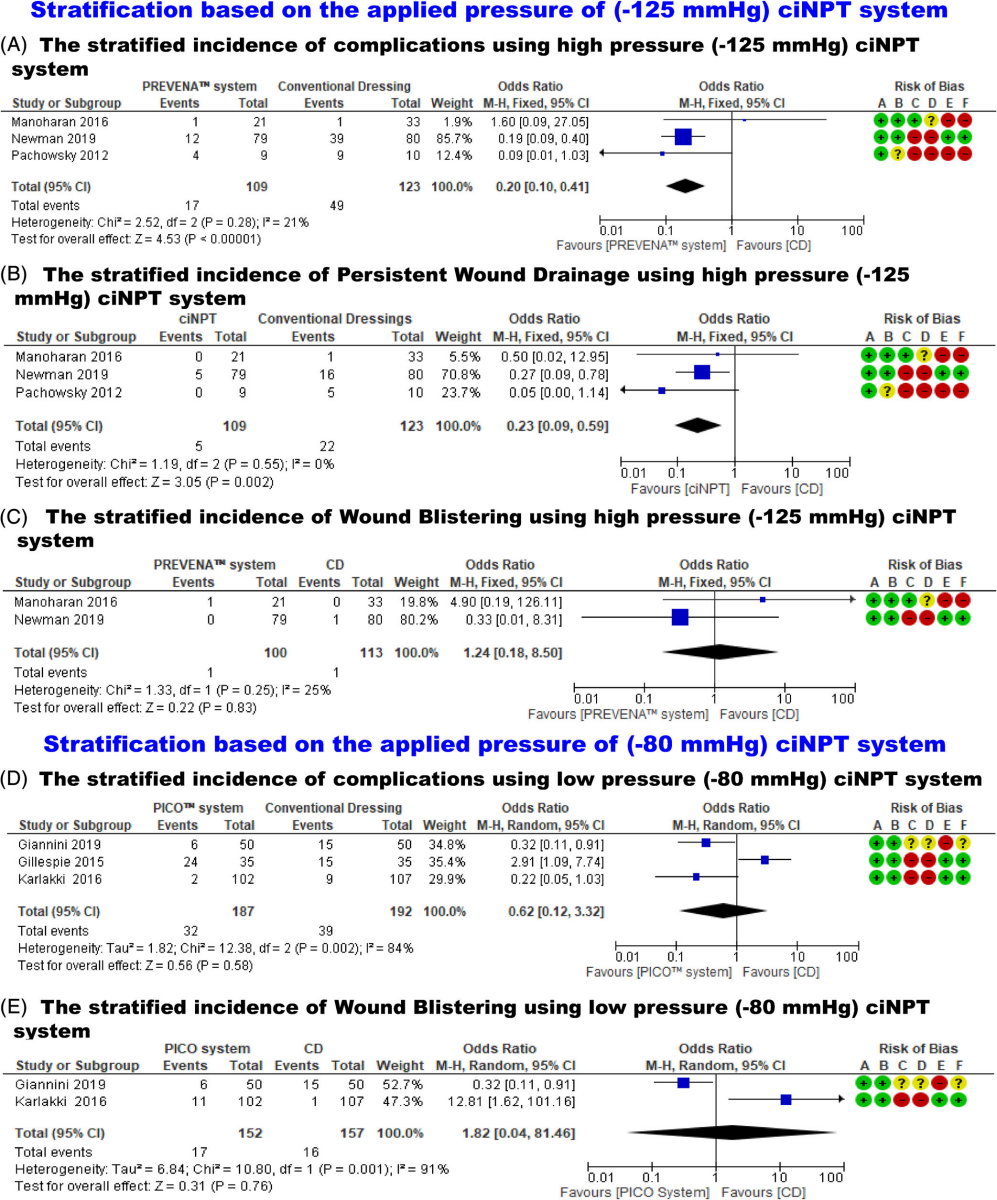
(A) Stratified incidence of complications using (−125 mmHg) closed‐incision negative pressure wound therapy (ciNPT), (B) stratified incidence of persistent wound drainage using −125 mmHg ciNPT, (C) Forest plots showing stratified incidence of wound blistering using −125 mmHg ciNPT, (D) stratified incidence of complications using (−80 mmHg) ciNPT, (E) stratified incidence of wound blistering using −80 mmHg ciNPT

Three studies,^21^, ^23^, ^24^ which included 232 patients, reported the incidence of complications using −125 mmHg ciNPT. There was no statistically significant heterogeneity in the studies (*P* = .28; *I* ^2^ = 21%). Using the fixed effects model, the outcome results revealed that the −125 mmHg ciNPT system had a statistically significant, lower risk of complications as compared to conventional dressings following total hip and knee arthroplasties (OR = 0.20; 95% CI: 0.10 to 0.41; *P* < .00001) (Table 4 and Figure 5A).

#### 4.2.2 Meta‐analysis of stratified incidence of persistent wound drainage using the −125 mmHg ciNPT system

Three studies,^21^, ^23^, ^24^ which included 232 patients, reported the incidence of persistent wound drainage using the −125 mmHg ciNPT system. There was no statistically significant heterogeneity in the studies (*P* = .55; *I* ^2^ = 0%). Using the fixed effects model, the results revealed that −125 mmHg ciNPT system use had a statistically significant lower risk of persistent wound drainage as compared to conventional dressing (OR = 0.23; 95% CI: 0.09 to 0.59; *P* = .002) (Table 4 and Figure 5B).

#### 4.2.3 Meta‐analysis of stratified incidence of blistering using the −125 mmHg ciNPT system

Two studies^21^, ^23^ which included 213 patients, reported the incidence of blistering using the −125 mmHg ciNPT system. There was no statistically significant heterogeneity in the studies (*P* = .25; *I* ^2^ = 25%). Using the fixed effects model, the outcome results revealed no statistically significant difference in the incidence of blistering between the −125 mmHg ciNPT system and conventional dressing (OR = 1.24; 95% CI: 0.18 to 8.50; *P* = .83) (Table 4 and Figure 5C).

#### 4.2.4 Meta‐analysis of stratified incidence of complications using the −80 mmHg ciNPT system

Three studies,^16^, ^20^, ^22^which included 379 patients, reported the incidence of complications using the −80 mmHg ciNPT system. There was statistically significant heterogeneity in the studies (*P* = .002; *I* ^2^ = 84%). Using the random effects model, the outcome results revealed that there was no statistically significant difference in the incidence of complications between the −80 mmHg ciNPT system and conventional dressing following total hip and knee arthroplasties (OR = 0.62; 95% CI: 0.12 to 3.32; *P* = .58) (Table 4 and Figure 5D).

#### 4.2.5 Meta‐analysis of stratified incidence of blistering using the −80 mmHg ciNPT system

Two studies,^16^, ^22^ which included 309 patients, reported the incidence of blistering using the −80 mmHg ciNPT system. There was statistically significant heterogeneity in the studies (*P* = .001; *I* ^2^ = 91%). Using the random effects model, the outcome results revealed no statistically significant difference in the incidence of blistering between the −80 mmHg ciNPT system and conventional dressing (OR = 1.82; 95% CI: 0.04 to 81.46; *P* = .76) (Table 4 and Figure 5E).

#### 4.2.6 Meta‐analysis of stratified incidence of complications following THA

**FIGURE 6:**
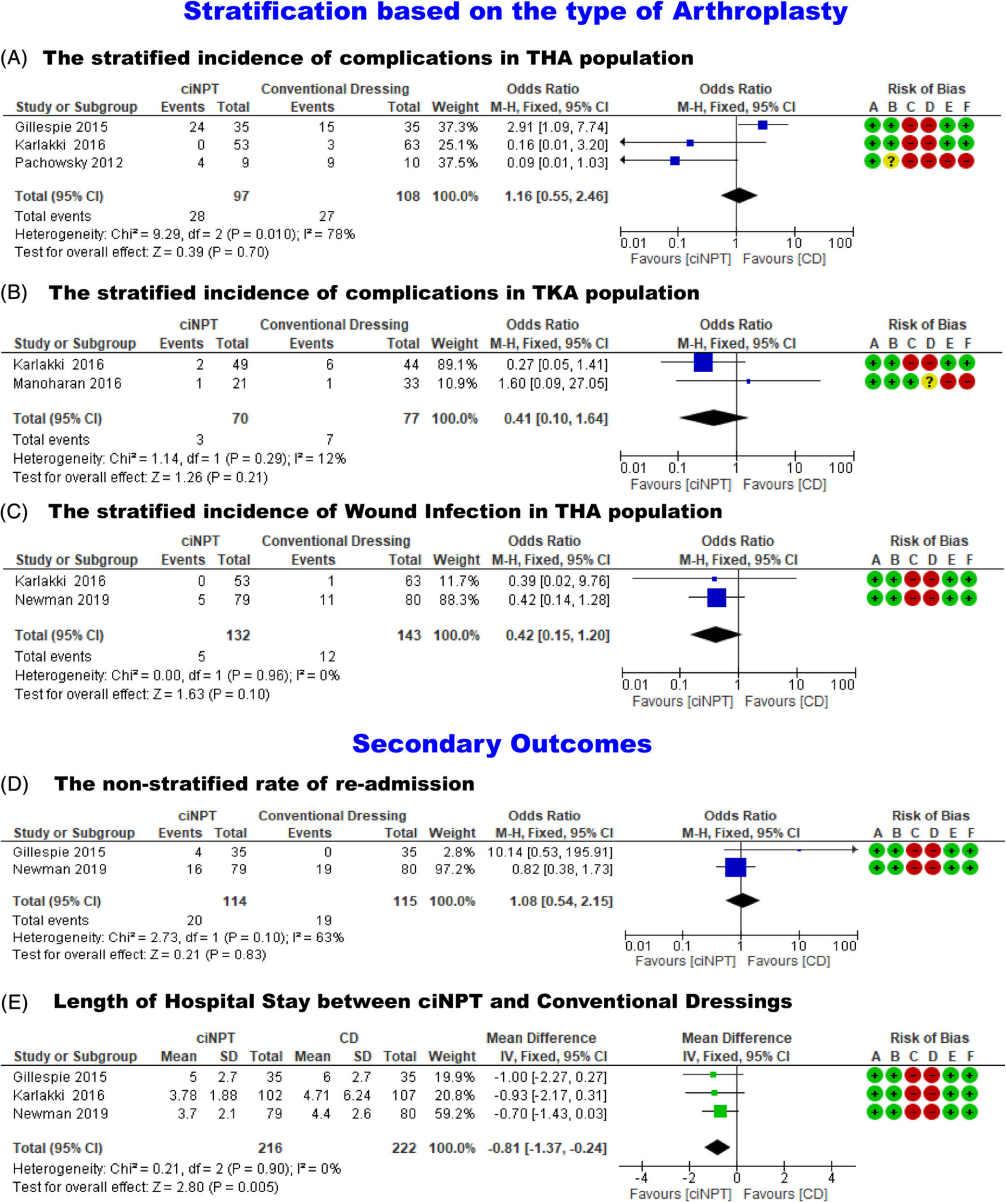
(A) Stratified incidence of complications in total hip arthroplasty (THA) population (B) stratified incidence of complications in total knee arthroplasty (TKA) population, (C) and stratified incidence of wound infection in THA population, (D) Forest plots showing non‐stratified rate of re‐admission (E) length of hospital stay between ciNPT and conventional dressings

Three studies,^16^, ^20^, ^24^ which included 205 patients, reported the incidence of complications following THA. There was no statistically significant heterogeneity in the studies (*P* = .010; *I* ^2^ = 78%). Using the fixed effects model, the outcome results revealed that there was no statistically significant difference in the incidence of complications between the ciNPT system and conventional dressing following THA (OR = 1.16; 95% CI: 0.55 to 2.46; *P* = .70) (Table 4 and Figure 6A).

#### 4.2.7 Meta‐analysis of stratified incidence of complications following TKA

Two studies^16^, ^23^ which included 147 patients, reported the incidence of complications following TKA. There was no statistically significant heterogeneity in the studies (*P* = .29; *I* ^2^ = 12%). Using the fixed effects model, there was no statistically significant difference in the incidence of complications between the ciNPT system and conventional dressing following TKA (OR = 0.41; 95% CI: 0.10 to 1.64; *P* = .21) (Table 4 and Figure 6B).

#### 4.2.8 Meta‐analysis of stratified incidence of wound infection following THA

Two studies,^16^, ^21^ which included 275 patients, reported the incidence of wound infection following THA. There was no statistically significant heterogeneity in the studies (*P* = .96; *I* ^2^ = 0%). Using the fixed effects model, there was no statistically significant difference in the incidence of wound infection between the ciNPT system and conventional dressing following THA (OR = 0.42; 95% CI: 0.15 to 1.20; *P* = .10) (Table 4 and Figure 6C).

## 5 SECONDARY OUTCOMES

### 5.1 Meta‐analysis of the readmission rate

Two studies,^20^, ^21^ which included 229 patients, reported the readmission rate. There was no statistically significant heterogeneity in the studies (*P* = .10; *I* ^2^ = 63%). The fixed effects model revealed that there was no statistically significant difference in the rate of readmission between ciNPT use and conventional dressing (OR = 1.08; 95% CI: 0.54 to 2.15; *P* = .83) (Table 4 and Figure 6D).

### 5.2 Meta‐analysis of length of hospital stay (LOHS)

Three studies^16^, ^20^, ^21^ reported the length of hospital stay in ciNPT and conventional dressings groups. There was no statistically significant heterogeneity in the studies (*P* = .90, *I* ^2^ = 0%). When the fixed effects model was used, the results indicated that the ciNPT group had a shorter length of stay (19.44 hours less) as compared to the conventional dressing group (MD = −0.81; 95% CI: −1.37 to −0.24; *P* = .005) (Table 4 and Figure 6E).

## 6 DISCUSSION

In this study, we performed a stratified and non‐stratified meta‐analysis of six RCTs. The stratified meta‐analysis was based on the pressure applied and on the type of surgery performed. To our knowledge, this is the first meta‐analysis investigating the efficacy of −125 mmHg ciNPT compared to −80 mmHg ciNPT and conventional dressing. The non‐stratified meta‐analysis showed a significant decrease in the incidence of wound drainage and LOS in patients treated with ciNPT compared to patients treated with conventional dressings. In contrast, no significant decrease in the incidence of wound complications in general, and, in particular, of infection, blistering, dehiscence, seroma, hematoma, and readmission was found. However, in several meta‐analyses, risks of developing a certain condition were lower, although not significant, such as in the case of wound complications and infections. Readmission rates were also lower compared to patients treated with conventional dressing. The stratified meta‐analysis showed that −125 mmHg ciNPT significantly decreased the incidence of wound complications and drainage compared to conventional dressings. Recently, it has been observed that the amount of drainage from wounds is a predictor of wound infections and prosthetic joint infection,^25^ suggesting that the application of −125 mmHg ciNPT might be consequential in preventing an environment favorable to bacterial growth. Requirements of a higher pressure for better outcomes were already shown in 2001, when Morykwas et al investigated the effect of negative pressures ranging from 0 to −400 mmHg with 25 mmHg increments. They found that maximum blood flow was achieved at −125 mmHg pressure.^26^ Higher pressures were found to be effective in keeping a drier wound environment due to stronger sucking action reaching deeper in the wounds.^27^ This is essential for certain types of hydrophilic dressings that are known to retain more liquids and have been shown to stabilize the wound and decrease pain.^28^, ^29^ Stratified meta‐analysis based on THA or TKA showed no statistically significant differences in the incidence of complications between ciNPT and conventional dressing following total hip or knee arthroplasties. Regarding wound infections, the stratified meta‐analysis did not show statistical significance.

Closed incision negative pressure therapy can be used over a variety of incisions and has been shown to help hold incision edges together, act as a barrier to external contamination, decrease lateral tension of sutured or stapled incisions, and reduce edema^30^ Some data are controversial because they are not consistent with a general improvement of SSC in using ciNPT even though a trend toward decreased infections is observed.^31^ A 2018 Cochrane study reporting three mortality studies, 25 SSI studies, and 14 dehiscence studies showed uncertainty regarding a significant benefit of ciNPT when compared to control‐treated patients.^32^ However, two recent meta‐analyses conducted by Singh et al in 2019 demonstrated that ciNPT usage was associated with statistically significant reductions in rates of SSIs as compared to conventional dressings.^33^, ^34^ Specifically, for the surgical procedure analysis that included THA, TKA, and hip and knee periprosthetic fracture surgery, the results showed a significant effect in favor of ciNPT over traditional dressings in reducing SSIs.^33^ Furthermore, the meta‐analysis from the second Singh et al study reported that patients in the control group were 3.17 times more likely to develop an SSI compared with patients in the ciNPT group.^34^ Therefore, the need for additional data and studies has arisen.

In orthopedics, as well as in other surgical interventions, the application of ciNPT was recommended by a panel analyzing the results of 100 publications between 2000 and 2015.^10^, ^13^ Several studies have shown the benefits of ciNPT in postoperative orthopedic surgery of patients undergoing primary elective hip and knee arthroplasties and revision surgeries including SSI, hospital stay, and healthcare cost reductions.^15^, ^16^, ^17^, ^35^ Data in the literature suggest that the average cost for ciNPT is about 10 times higher than conventional dressings.^20^, ^23^ Our results confirmed a shorter length of stay for patients treated with ciNPT, which may balance the more expensive treatment, as suggested in the literature.^36^

One potential complication of ciNPT use is the formation of skin blisters, usually found in the peri‐wound skin away from the surgical incision.^15^, ^22^, ^37^ This may be related to improper application technique and friction of negative pressure dressing on skin.^15^, ^38^ Blisters are more common in patients undergoing TKA^37^, ^38^ and with the application of polyurethane foam.^22^, ^37^ Efforts have been aimed at developing a new kind of silicone foam to decrease blister formation in TKA.^22^ Our meta‐analysis did not find any significance association between blister formation and ciNPT use compared to conventional dressings, regardless of the pressure applied, suggesting that a stratified analysis may give more reliable and specific results. A meta‐analysis by Awad et al investigating published clinical trials on autologous bone marrow mesenchymal stem cells in the repair of cartilage lesions of the knee has proposed recommendations and guidelines for the required data to be reported in future clinical trials.^39^ The authors proposed guidelines to the orthopedic research community to assure repeatability and reproducibility of data. We propose that similar consistent guidelines should be implemented in clinical trials investigating ciNPT, such as study design, patient characteristics, type of surgery, type of ciNPT application, modality of the application, and postoperative protocol.

There are limitations to consider regarding this study. There were inherent limitations within the current literature that compromised our analyses. The literature lacks clinical studies that specifically compare the two pressure systems to each other. Further RCTs comparing both systems are required to make a clinically appropriate choice between −125 mmHg and −80 mmHg ciNPT. The data may be heterogeneous, including demographic data, conventional dressing applied, and follow‐up information. The sample size becomes relatively small when the stratified analysis is performed. Consistency is another limitation ‐ our data are not stratified by revision or primary surgery and patients are not divided according to their comorbidities, both of which have been shown to have a significant effect on complications.^30^ There was potential for bias in analyses deriving large proportions of their sample size from a single study. In addition, each individual study contained biases which may have affected this study's outcomes, including attrition bias, reporting bias, detection bias, and performance bias.

## 7 CONCLUSION

Comparing outcomes in postoperative orthopedic patients in a stratified and non‐stratified meta‐analysis, our findings suggest that ciNPT displayed great benefits for patient quality of life and healthcare cost. The stratified meta‐analysis indicated that patients undergoing treatment with −125 mmHg ciNPT displayed significantly fewer overall complications and persistent wound drainage when compared to conventional dressings, suggesting that −125 mmHg ciNPT might prevent infections at a higher rate. Thus, it is recommended that orthopedic surgeons utilize −125 mmHg ciNPT for postoperative wound care in patients undergoing THA or TKA. Future studies that account for comorbidities, have greater sample sizes, and stratifiy by revision or primary surgery may be beneficial to the literature.

## CONFLICT OF INTEREST

Dr Saleh serves as a paid consultant for Aesculap/B.Braun, CONMED Linvatec, 3 M‐KCI foundation, and Ranfac Corp. Other co‐authors have no conflict of interest to disclose.

## AUTHOR CONTRIBUTION

Conceptualization: Furqan Irfan, Joshua Lumbley, Gamal Mostafa, Khaled J Saleh

Data Curation: Kareem Elhage, Mohamed Awad

Formal Analysis: Kareem Elhage, Mohamed Awad

Investigation: Furqan Irfan, Joshua Lumbley, Gamal Mostafa, Khaled J Saleh

Project Administration: Furqan Irfan, Joshua Lumbley, Gamal Mostafa, Khaled J Saleh

Supervision: Furqan Irfan, Joshua Lumbley, Gamal Mostafa, Khaled J Saleh

Writing – Original Draft Preparation: Kareem Elhage, Mohamed Awad

Writing – Review & Editing: Furqan Irfan, Joshua Lumbley, Gamal Mostafa, Khaled J Saleh

## TRANSPARENCY STATEMENT

The authors affirm that this manuscript is an honest, accurate, and transparent account of the study being reported; that no important aspects of the study have been omitted; and that any discrepancies from the study as planned (and, if relevant, registered) have been explained. The contents of this systematic review do not neither include any data from nor represent the views of the Department of Veterans Affairs or the United States Government.
